# Correlation Between Thrombin Generation and Hepatic Fibrosis in Chronic Liver Diseases

**DOI:** 10.7759/cureus.71376

**Published:** 2024-10-13

**Authors:** Liliana Vecerzan, Ionela Maniu, Gabriela Cioca

**Affiliations:** 1 Pharmacology, Emergency Military Clinical Hospital, Sibiu, ROU; 2 Faculty of Medicine, Lucian Blaga University, Sibiu, ROU; 3 Mathematics and Informatics Department, Research Center in Informatics and Information Technology, Faculty of Sciences, Lucian Blaga University, Sibiu, ROU; 4 Pharmacology, Clinical County Hospital of Emergency, Sibiu, ROU; 5 Clinical Studies and Drug Safety Department, Faculty of Medicine, Lucian Blaga University, Sibiu, ROU

**Keywords:** coagulation disorders, fibrosis, hepatitis virus, liver cirrhosis, thrombin generation

## Abstract

Hepatic stellate cells (HSC) are recruited to the site of injury in the liver tissue repair mechanism, and the changes HSC undergo reflect paracrine stimulation by all cell types (sinusoidal endothelial cells, Kupffer cells, hepatocytes, platelets, and leukocytes). This study aimed to analyse a possible correlation between the degree of liver fibrosis and thrombin generation in patients with chronic liver diseases.

Background

Coagulation disorders in patients with chronic liver disease continue to challenge clinicians. A more accurate assessment of the coagulation cascade is provided by investigating thrombin production in individuals with chronic liver disease with varying degrees of fibrosis.

Methods

This prospective observational pilot study was done on hospitalized patients with chronic liver diseases. Thrombin generation from their platelet-poor plasma was studied and the degree of liver fibrosis assessed by transient elastography versus that of control subjects.

Results

The peak thrombin is significantly higher in those with severe liver stiffness (F3) than in those with more advanced fibrosis/liver cirrhosis (F4). Also, the total concentration of thrombin formed in the time interval (area under the curve (AUC)) and velocity index (VI) is higher in those with F3 than those with F4.

Conclusion

Thrombin generation decreases with the progression of liver fibrosis.

## Introduction

Thrombin represents a serine protease with an enzymatic role, mainly in hemostasis, but its versatile action was described recently in endothelium metabolism as it favours inflammatory changes and angiogenesis [1]. The pleiotropic action of thrombin was found to an extent on platelets, monocytes, and lymphocytes, and it is involved in tissular reparation through protease-activated receptors (PARs) [2]. PAR stimulation is one of the mechanisms through which thrombin influences cellular defence and apoptosis [2]. Nevertheless, the liver is directly involved in haemostasis due to its synthesis function, especially with respect to coagulation factors and proteases alongside the inflammatory molecules. Consequently, compromised liver function is associated with both hypercoagulability and bleeding disorders due to an abnormal balance of pro- and anticoagulant plasma factors [3]. In the mechanism of liver tissue repair, hepatic stellate cells (HSCs) are recruited to the site of injury, and the changes they undergo reflect paracrine stimulation by all types of cells (sinusoidal endothelial cells, Kupffer cells, hepatocytes, platelets, and leukocytes).

Thrombin converts circulating fibrinogen into fibrin, promotes platelet aggregation, acts as a potent stimulator of endothelial cells, serves as a chemotactic factor for inflammatory and fibroblastic cells, and is mitogenic for vascular smooth muscle cells. The cellular effects of thrombin are mediated by the G protein-coupled receptors, which are PARs. All known members of the PAR family stimulate the proliferation/activation of HSCs. [4]

Thrombin receptors are constitutively expressed in the liver, and their expression increases in parallel with the severity and/or duration of the liver disease. Studies in humans have been conducted on thrombotic risk factors, which have been independently associated with the extent of fibrosis. Some studies [5] have shown that anticoagulants or antiplatelet agents prevent hepatic necrosis and fibrosis by acting on HSCs. These drugs could be considered therapeutic agents in patients with chronic liver conditions, and specific studies should be initiated to explore them further. Liver fibrogenesis is a dynamic process involving complex cellular and molecular mechanisms, leading to chronic activation of liver tissue repair mechanisms [5]. One of the first steps in tissue repair is recruiting inflammatory cells to neutralize potentially infectious agents and remove necrotic tissue. In this phase of the process, HSC, as well as other cells of the extracellular matrix (e.g., fibroblasts and myo-fibro/blasts), are recruited to the site of injury and undergo an activation process leading to a phenotype characterized by increased property proliferative, motility, and contractility [6]. Liver stellate cell changes reflect paracrine stimulation of all neighbouring cell types, including sinusoidal endothelium, Kupffer cells, hepatocytes, platelets, and leukocytes [7]. Platelets are the first cells recruited to the injury site, playing an essential role in healing processes, as they limit blood loss by forming aggregates in injured blood vessels. Moreover, they act as a platform for forming fibrin from fibrinogen. In addition, their alpha granules are rich in growth factors such as platelet-derived growth factor (PDGF), transforming growth factor-beta (TGFb), and vascular endothelial growth factor (VEGF), which are stimulators of fibroblasts and other mesenchymal cells, important in the tissue healing process [8]. Preclinical studies showed that in the tissue-repairing process and remodelling, the thrombin receptor PARs are linked to the bradykinin receptors-β 2, which are up-regulated in hepatic fibrogenesis and exert an immune function in the hepatic damaging process. [9,10]. Moreover, in chronic liver impairment, the increased activity of thrombin, alongside factor Xa, was found to be a trigger for the up-regulated PARs related to the occurrence and progression of hepatic fibrosis [10]. The process of fibrinolysis intervenes as a protection mechanism against thrombotic phenomena and thrombi occurrence. This process involves a circle of inactive precursors that are activated (such as plasminogen into plasmin). On the other hand, anti-fibrinolytic agents are engaged (such as plasminogen activator inhibitor or thrombin activable fibrinolysis inhibitor) [11].

## Materials and methods

We conducted a prospective observational pilot study between January 2017 and March 2018 that included 59 patients with chronic liver diseases admitted to the gastroenterology and internal medicine wards at the Sibiu County Emergency Clinical Hospital. All subjects agreed to participate and signed informed consent (World Medical Association Code of Ethics, Declaration of Helsinki). The hospital's ethical committee approved the study, ensuring patient data confidentiality. The criteria for including subjects in the study were age equal to or older than 18 years, signing the study participation agreement, hospitalization for chronic liver diseases in various evolutionary stages, and paraclinical evaluations, including thrombin generation and FibroScan (Echosens, Paris) examination. The exclusion criteria were age younger than 18 years, subjects with ascites (which excludes FibroScan examination), subjects in whom thrombin generation could not be determined, the presence of gastrointestinal bleeding with hemodynamic instability, shock or coma of different aetiologies, acute cardiovascular diseases (acute coronary syndrome, recent stroke), chronic or chronically exacerbated cardiovascular diseases in treatment with antiplatelet agents, anticoagulants, fibrinolytics and with low-performance status (Eastern Cooperative Oncology Group (ECOG) 4), current treatment with fresh frozen plasma or Vitamin K, neoplasias with extrahepatic locations, any acute or chronic infections or inflammations.

A blood sample was collected from each subject into 4.5 ml CTAD (citrate-theophylline-adenosine-dipyridamole, Vacutainers®, Beckton Dickinson, Franklin Lakes, NJ) glass tubes containing 3.2% 0.109 M sodium citrate. Thrombin generation was performed from platelet-poor plasma using the fully automated Ceveron alpha TGA Tech-nothrombin® reagent kit (Technoclone, Vienna, Austria).

The results were automatically calculated by Technothrombin® TGA (Technoclone, Vienna, Austria) for Ceveron® alpha fully automated software (DiaPharma Group Inc., West Chester Township, OH), which provided the following parameters: Lag time (phase) (min), measured from the moment of adding the tripterygium glycoside tablet (TGT) reagents until the first generation of thrombin (first burst); Peak Thrombin (nM), the maximum thrombin concentration generated; Time to peak (min), the time required to reach the maximum concentration of thrombin produced; Velocity index or slope (nM/min) = [Peak height/(time to peak − lag time)] - slope inclination; The area under the curve (AUC) (nM), the total concentration of thrombin formed in the time interval, a parameter also known as endogenous thrombin potential (ETP). This curve integrates all procoagulant and anticoagulant reactions and thrombin formation and inhibition, comprising the coagulation cascade's initiation, propagation, and terminal phase. It is obtained from the fluorescent signal that develops when plasma coagulates in the presence of the fluorogenic substrate for thrombin. The Ceveron® alpha automatic analyser has four measuring channels with a fluorometric module for TGT consisting of an ultraviolet light-emitting diode (UV LED) (365nm) for excitation and a photodiode for measuring the emitted signal, placed in the rotor of the cuvettes (thermostat), according to the study carried out by Olteanu et al. [12,13].

The degree of liver fibrosis was assessed by transient elastography (FibroScan), a non-invasive method for determining the elasticity of the liver tissue. This evaluation is a quick, painless method that does not require sedation. This technique uses a special probe generating vibration waves of 50 Hz frequency and 2 mm amplitude. The result is expressed in kiloPascals (kPa) and is the median value of at least 10 measurements. The values obtained from this investigation are reproduced in Table 1 [14].

**Table 1:** Suggested liver stiffness values for fibrosis staging This table outlines the progression of liver stiffness measured in kilopascals (kPa) as fibrosis and cirrhosis advance. Mild stiffness (F≥1) indicates early-stage fibrosis (minimal scarring); significant stiffness (F≥2) is associated with moderate liver fibrosis; severe stiffness (F≥3) reflects advanced liver fibrosis but not yet cirrhosis; liver cirrhosis (F=4) indicates full-blown cirrhosis with extensive scarring [14].

Liver stiffness	Value (kPa)
Mild stiffness (F≥1)	4.9-5.3
Significant stiffness (F≥2)	6.8-7.4
Severe stiffness (F≥3)	8.6-9.1
Liver cirrhosis (F=4)	11.8-13.6

For each evaluated parameter, the score is established according to Table 2. The individual scores are summed to yield the following Child-Pugh stages [15].

**Table 2:** The Child-Pugh stages Child-Pugh classification is obtained by adding a score for each parameter [15]: Class A = 5 to 6 points (least severe liver disease) Class B = 7 to 9 points (moderately severe liver disease) Class C = 10 to 15 points (most severe liver disease)

Clinical and Lab Criteria	A	B	C
Ascites	None	Mild	Severe
Encephalopathy	None	Mild	Severe
Bilirubin (mg/dL)	2	2-3	3
Albumin (g/%)	3.5	2.8-3.5	2.8
International Normalised Ratio (INR)	<1.7	1.7-2.3	>2.3

## Results

The patient population's demographic characteristics and laboratory data are summarized and presented in Tables 3, 4.

**Table 3:** Demographic characteristics of patients BMI= body mass index, HBV=hepatitis virus B, HCV=hepatitis virus C; IQR: interquartile range.

Characteristic		Child-Pugh A (n = 16)	Child-Pugh B (n = 19)	Child-Pugh C (n = 5)	p
Sex n (%)	M	7 (43.75)	11 (57.89)	3 (60.00)	0.904
	F	9 (56.25)	8 (42.11)	2 (40.00)	
Age (median (IQR))		62 (44; 80)	65 (43; 87)	61 (56; 70)	0.887
BMI (median (IQR))		28.58 (21.45; 35.98)	28.58 (20.98; 33.20)	28.52 (24.69; 29.76)	0.933
Aetiology (n (%))					0.132
HBV		0 (0.00)	2 (10.53)	1 (20.00)	
HCV		9 (56.25)	3 (15.79)	0 (0.00)	
Alcohol		7 (43.75)	11 (57.89)	3 (60.00)	
Viral+Alcohol		0 (0.00)	2 (10.53)	1 (20.00)	
Others		0 (0.00)	1 (5.26)	0 (0.00)	
Smokers (n (%))		9 (56.25)	7 (36.84)	2 (40.00)	0.491
Ethanol consumer (n (%))		7 (43.75)	11 (57.89)	2 (40.00)	0.740
Cardiovascular diseases (n (%))		6 (37.50)	8 (42.11)	0 (0.00)	0.232
Diabetes mellitus (n (%))		4 (25.00)	8 (42.11)	1 (20.00)	0.638
Renal disease (n (%))		2 (12.50)	0 (0.00)	0 (0.00)	0.243

In both hepatitis and cirrhosis groups, more than 40% of the patients were smokers. The median age of the control group was 45 years (IQR: 40; 73). In this group, 75% were female, and the median body mass index (BMI) for the group was 23.51 (IQR: 17.26, 39.33). Nineteen (32%) patients in the study group had chronic hepatitis and 40 (68%) patients had liver cirrhosis. The aetiology of chronic liver diseases included hepatitis viruses (virus C - 45.76%, virus B - 8.47%), ethanol (38.98%), and an association between hepatitis viruses (5.08%) and other aetiologies (1, 69%).

**Table 4:** Laboratory data according Child-Pugh Score Values represent median (IQR (interquartile range) P25–P75). AST: aspartate aminotransferase; ALT: alanine aminotransferase; TB: total bilirubin; DB: direct bilirubin; AL: alkaline phosphatase; GGT: gamma-glutamyl transpeptidase; T prot: total protein

Parameters	Child-Pugh A (n = 16)	Child-Pugh B (n = 19)	Child-Pugh C (n = 5)	p
AST(U/L)	35 (16; 190)	43 (19; 247)	55 (28; 127)	0.382
ALT (U/L)	32.50 (7; 166)	24 (8; 115)	34 (17; 68)	0.963
TB (mg/dL)	0.88 (0.38; 11.74)	2.19 (0.72; 15.92)	4.33 (2.52; 18.85)	0.000
DB (mg/dL)	0.57 (0.47; 8.21)	1.54 (0.71; 14.17)	2.77 (1.68; 16.38)	0.036
AL (U/L)	82 (51; 345)	125 (48; 286)	83 (52; 168)	0.073
GGT (U/L)	49 (19; 176)	88 (15; 1437)	50 (10; 185)	0.218
T prot(g/dL)	7.50 (6.40; 9.30)	7 (5.40; 9.00)	5.40 (4.80; 7.60)	0.015
Albumin (%)	52.15 (33.20; 60.30)	43.50 (29.40; 58.70)	47 (29.60; 54.20)	0.013
Gamma-globulin (%)	22.45 (13.50; 42.00)	27.85 (14.70; 45.40)	33 (18.60; 48.60)	0.049

The parameters of thrombin generation were grouped according to the stages of liver fibrosis (Table 5). The peak parameter is significantly higher in those with F3 (median 295.5, IQR: 203.30-341.20) compared to those who have a more advanced degree of fibrosis (F4) (median 175, IQR: 132.90-228.10; p<0.05); also area under the curve (AUC) and velocity index (VI) (the latter approaches statistical significance) are higher in those with F3, compared to those with F4 (p<0.05).

**Table 5:** Parameters of thrombin generation according to stages of liver fibrosis Quantitative variables are expressed as median (IQR (interquartile range)); tPeak=peak time, Peak=peak thrombin, VI=velocity index; AUC=area under the curve, F2=fibrosis stage 2, F3=fibrosis stage 3, F4=fibrosis stage 4.

Parameters of thrombin generation	F2	F3	F4	p
Lag phase-0	3.05 (2.70;3.40)	3.70 (2.50;4.10)	3.80 (3.40;4.30)	0.098
tPeak (min)-0	6.00 (5.20;6.80)	6.90 (5.50;8.20)	7.55 (7.00;8.50)	0.105
Peak (nM)-0	256.15 (206.90;305.40)	295.50 (203.30;341.20)	175.00 (132.90;228.10)	0.022
VI (nM/min)-0	72.65 (60.70;84.60)	85.60 (47.50;122.20)	45.95 (33.40;65.00)	0.053
AUC (nM)-0	2279.05 (1864.20;2693.90)	2427.90 (2191.30;2669.70)	1974.30 (1613.40;2236.00)	0.044

Another score proposed to indicate the degree of severity of liver diseases is the Child-Pugh score. In our study, we tried to demonstrate if there is a possible correlation between the Child-Pugh score and the parameters of thrombin generation. The Child-Pugh score is inversely correlated with the parameters Peak (rho=-0.596, p=0.000), VI (rho=-0.667, p=0.000), and AUC (rho=-0.579, p=0.000). The results obtained are highlighted in Table 6.

**Table 6:** Correlation between Child-Pugh score and parameters of thrombin generation *Significance level p≤0.05; ** significance level p≤0.005. tPeak=peak time, Peak=peak thrombin, VI=velocity index; AUC=area under the curve

Spearman's rho		Lag phase	tPeak (min)	Peak (nM)	VI (nM/min)	AUC (nM)
	Correlation coefficient	0.202	0.286	-0.596**	-0.667**	-0.579**
Child-Pugh	Level of significance	0.210	0.073	0.000	0.000	0.000
	N	40	40	40	40	39

## Discussion

In fibrotic stages, chronic liver disease is accompanied by changes in properties and quantitative synthesis of the proteins involved in the coagulation cascade. Multiple factors influence the dysregulation of these mechanisms, including a decreased release of thrombopoietin, which is engaged in the maturation process of the platelets. In the cirrhotic liver, the imbalanced protein and protease synthesis contribute to thrombotic events alongside portal hypertension, which favours portal vein thrombosis and variceal bleeding [16]. The evaluation and accurate assessment of the coagulation state in cirrhotic patients might be clinically challenging due to limitations of prothrombin time and international normalized ratio [17]. In this regard, liver disease is associated with a complex coagulopathy that is prone to thrombosis and bleeding [18], and thrombin testing was proposed to be used in conjunction with soluble thrombomodulin. This should alleviate the influence of protein anticoagulant action [18]. Moreover, coagulopathy mirrors the extent of hepatic function damage, as prolonged prothrombin time indicates a poor prognosis in acute liver failure, while in liver cirrhosis staging, prothrombin time is utilized for calculating the Child-Pugh score. Although coagulation tests are abnormal in chronic liver disease, bleeding events happen mainly through variceal bleeding due to poor synthesis of anticoagulant factors in the insufficient liver [19]. Notably, thrombin stimulates the expression of plasminogen activator inhibitor-1 (PAI-1) at the endothelial level, PAI-1 having an important bio-function in the fibrinolytic process. Even more, in the study of Huebner et al., thrombin induced a significant PAI-1 expression in hepatic vascular endothelial cells compared to those from the lungs. The authors suggested that hepatic endothelial cells possess pre-synthetized PAI-1 and might participate more in PAI-1 synthesis. [20]. The rationale was that therapeutic intervention for a hypercoagulable state should aim at the liver [20].

This study shows significant differences in thrombin generation depending on the degree of liver fibrosis. Patients with a more advanced degree of liver fibrosis showed a significantly higher Lag phase parameter, while the Peak, VI, and AUC parameters were significantly lower in patients with F4 fibrosis compared to those in the F3 group; it can be concluded that thrombin generation decreases not only with chronic liver disease and the liver fibrosis stage progression but also with increasing Child-Pugh stage (of patients with liver cirrhosis). In the literature, the complexes between thrombin and antithrombin were increased with the severity of chronic liver disease. In contrast, free-activated factor XII and antithrombin levels decreased in the plasma of cirrhotic patients. The same study reported that an increased thrombin generation was not associated with an observable activation of intrinsic or extrinsic coagulation pathways [21].

The results obtained in our study are similar to those obtained by Dillon et al. [22], who performed an analysis in which they included 61 patients with compensated liver cirrhosis. They studied the characterization of thrombin generation in patients with Child-Pugh A liver cirrhosis and observed that coagulation parameters change during liver cirrhosis. Tissue factor-stimulated thrombin generation was measured by calibrated automated thrombography, and the degree of liver fibrosis was assessed by transient elastography. The results showed that with the increase in the degree of liver fibrosis, the propagation phase, attenuation phase, and thrombin generation significantly decreased. The degree of liver fibrosis is correlated with ETP, as well as the propagation and attenuation phases. This study concluded that changes in thrombin generation associated with the degree of liver fibrosis during the evolution of liver cirrhosis can be considered clinical markers of transition from compensated to decompensated liver cirrhosis [22]. In the presented study, ETP was significantly lower in patients with fibrosis grade F4 compared to those with F3; the same results were obtained by Brodard et al. [23] in a study in which they enrolled 78 patients with chronic liver diseases (advanced chronic liver disease (ACLD)), where they studied whether parameters of thrombin generation correlate with liver stiffness. Liver stiffness was measured by transient elastography using 13 and 21 kPa as cutoffs (liver stiffness of ≥13 kPa but ≤21 kPa was associated with the diagnosis of cirrhosis) and thrombin generation was assessed by the ST Genesia® Thrombin Generation System (STG) using Thromboscreen with or without thrombomodulin (TM) and calibrated automated thrombography (CAT) using low PPP reagent (5 pmol/L TF and 4 pmol/L phospholipids) (Stago, Asnières sur Seine Cedex France) with or without TM. ETP inhibition by TM was more significant in patients with chronic liver disease with LSM <13 kPa than in those with LSM ≥13 but <21 kPa, indicating a lower sensitivity to TM in patients with liver stiffness values ≥13 but <21 kPa. In patients for whom CAT was performed, ETP was a measure of thrombin generation and with the addition of thrombomodulin, it was expressed as the normalized ratio. ETP inhibition by TM was more significant in patients with acute or chronic liver disease with liver stiffness <13 kPa than those with ≥13 but <21 kPa, as well as the group with liver stiffness ≥21 kPa, indicating a lower sensitivity to TM in patients with liver stiffness values ≥13 kPa. However, there was no difference in ETP inhibition by thrombomodulin between the group with liver stiffness ≥13 but <21 and the group with liver stiffness ≥21 kPa. In addition, thrombin generation was measured by both STG and CAT in 35 patients with acute or chronic liver diseases, which is a comparison of the two methods. Comparing our study with this one, we found lower ETP values in patients with F4 fibrosis (greater than 11.8 kPa) than in F3 (less than 9.1 kPa). Still, we used a different measurement method of thrombin generation and we did not use thrombomodulin [22,23]. Thrombin generation decreases not only as chronic liver disease progresses but also as the stage of liver fibrosis of patients with chronic liver disease and the Child-Pugh stage (of patients with liver cirrhosis) increases in our study, as it inversely correlates with parameters of thrombin generation: Peak, VI, and ETP. Among the non-invasive ways to explore liver fibrosis, the degree of liver fibrosis estimated using FibroScan correlated best with thrombin generation parameters. The results of our study demonstrated an inverse correlation between the Child-Pugh class and the Peak, VI, and ETP parameters. The explanation would be the following: the more the liver cirrhosis progresses (expressed by the Child-Pugh class), the lower the thrombin generation parameters (Peak, VI, ETP). Our results are similar to those obtained by Zermatten et al. [24] where they showed that ETP without thrombomodulin was significantly lower in patients with liver cirrhosis compared to the control group. They highlighted the trend of decreased ETP in the absence of thrombomodulin along with increasing Child-Pugh score, which reached statistical significance only when comparing Child-Pugh Class A with C and B with C [24,25]. Moreover, in studies including patients with direct-acting antivirals, the treatment stabilizes this balance, making it less likely to be disrupted than before when both pro- and anticoagulants were partially deficient. [26,27].

Another study with results similar to ours was performed by Tripodi et al. [28,29]; they classified the severity of liver disease according to the Child-Turcotte-Pugh staging, where they expressed the results of thrombin generation in ETP (FU /min), with or without the addition of thrombomodulin for patients and controls. Without the addition of thrombomodulin, the median ETP was significantly lower in patients than in controls, indicating that less thrombin was generated in cirrhotic patients. ETP values in patients correlated negatively with the Child-Turcotte-Pugh class [28, 29].

In our study, the aetiology of cirrhosis was viral and alcohol-induced. Still, out of 15 patients with viral aetiology cirrhosis, 12 (75%) had HCV infection. The hepatitis C virus affects the mechanisms involved in the coagulation cascade. In HCV infection, the endothelial cells exposed to the viral genome will react by an inflammatory response expressed by tumour necrosis factor (TNF) release. The viral ribonucleic acid (RNA) acts as a ligand for endothelial toll-like receptors-3 (TLR-3). The second mechanism through which HCV promotes endothelial injury is cryoglobulinemia, which is consecutively followed by the development of circulant immune complexes that propagate the inflammation forward. These pathways induce a hypercoagulable state and decrease thrombomodulin release. Virus infections might depress the normal anticoagulant pathways by inhibiting the production of thrombin via tissue factor and the fibrinolysis process [30].

Our study's limitations are the small sample size and the fact that it is monocentric. Another limitation might be that the elastographic determinations were made with the same FibroScan but by different observers, and there is the possibility of differences in the interpretation of the measurements. The strength of our study is the correlation of thrombin generation with the severity of cirrhosis, as assessed by the Child-Pugh score.

In another study by Gatt et al., thrombin generation assays proved to be more feasible in assessing the hypercoagulable state in patients with advanced liver disease. In this study, the ETP and velocity of thrombin generation were not correlated with INR, and the velocity of thrombin generation was higher in advanced stages of liver disease when INR values varied between 1.2 and 2.0 [31]. Moreover, Gatt et al. concluded that prothrombin time and INR are not recommended as markers of haemorrhagic risk for these patients [31].

The importance of haemostatic condition in chronic liver disease derives from the delicate balance between impaired clotting associated with haemorrhagic risk in a disease that combines the deficit of anticoagulant and pro-thrombotic status.

## Conclusions

The study's findings demonstrate a notable decrease in thrombin generation parameters (Peak, VI, AUC) as liver fibrosis and cirrhosis advance. Patients with more severe liver fibrosis (F4) exhibit lower thrombin generation than those with milder fibrosis (F3). Specifically, the peak thrombin, velocity index (VI), and area under the curve (AUC) values are significantly reduced in F4 patients, reflecting diminished thrombin production as liver disease worsens. Moreover, there is an inverse correlation between the Child-Pugh score, which evaluates liver disease severity, and thrombin generation parameters. This implies thrombin generation declines as the Child-Pugh score rises, indicating a more advanced cirrhosis. These results suggest that thrombin generation parameters, particularly Peak, VI, and AUC, could serve as valuable markers of liver disease progression and aid in assessing the shift from compensated to decompensated cirrhosis.

In summary, thrombin generation decreases significantly with advancing liver fibrosis and cirrhosis, correlating with the Child-Pugh score, which may be useful in monitoring the severity of liver disease. The more advanced the Child-Pugh class, the greater the decrease in thrombin generation, as reflected in Peak thrombin, VI, and AUC values, which are inversely correlated with the Child-Pugh class. These findings highlight the potential of thrombin generation parameters to serve as indicators of liver disease progression.
